# Consistency of Ultrasound Measurements of Fat Thickness in Different Postures

**DOI:** 10.2174/0115734056369864250725081342

**Published:** 2025-07-30

**Authors:** Yang Gao, Xinyi Tang, Min Li, Li Qiu

**Affiliations:** 1 Department of Medical Ultrasound, West China Hospital of Sichuan University, No. 37 Guoxue Alley, Chengdu 610041, Sichuan, China

**Keywords:** Ultrasound, Fat thickness, Consistency, Posture, Obesity, Correlation analysis, Intra-observer consistency

## Abstract

**Introduction:**

Ultrasound has been used in the field of clinical nutrition to measure body composition. However, the consistency of these measurements varies across studies, and the impact of examination posture remains largely unexplored, creating a critical methodological gap in clinical practice. The purpose of this study was to investigate the consistency of ultrasonic measurement of fat thickness (FT) and evaluate the impact of posture on these measurements.

**Methods:**

FT was measured at 10 body sites in routine and special postures using ultrasound to determine intra-observer and inter-observer consistency and to assess the impact of different postures on FT measurements. Body fat mass (BFM) was measured by bioelectrical impedance analysis (BIA), and subcutaneous skinfold thickness was measured with calipers for correlation analysis.

**Results:**

Results revealed significant sex differences in BFM (P<0.05) and FT at most sites (P<0.001), with women exhibiting thicker fat measurements. High intra-observer and inter-observer consistency was demonstrated in special examination postures (intraclass correlation coefficients were both ≥0.925). Posterior upper arm FT measured in the sitting posture was greater than that measured in the prone posture (P<0.001) while there was no significant difference in subscapular FT between the two postures (P = 0.289).

There were significant differences in posterior lower leg FT among the four postures (P<0.001). Positive correlations were observed between FT and skinfold at site 5 (abdominal subcutaneous fat), site 7 (posterior upper arm), and site 8 (subscapular) (r = 0.921, 0.878, 0.882, P<0.01).

**Discussion:**

Ultrasound measurements of FT have proven reliable, offering advantages in cost, ease, accuracy, and scalability. The findings highlight the importance of posture in ultrasound measurement of FT, which may influence clinical practice and research protocols. The limitations of the study mainly lie in the narrow age and BMI ranges of the sample, which restrict the generalizability of the research findings.

**Conclusion:**

This study provides a comprehensive evidence base for posture-specific ultrasound protocols in fat thickness measurement. Our results demonstrate that ultrasound is a reliable method for measuring fat thickness, exhibiting good to excellent inter-observer and intra-observer consistency. The impact of body posture on fat thickness measurements varies by anatomical location. Strong correlations were found between ultrasound measurements and skinfold thickness at subcutaneous sites, confirming the validity of ultrasound for fat thickness assessment.

## 1 INTRODUCTION

Malnutrition, characterized by inadequate, excessive, or imbalanced energy and nutrient intake, is a major global health concern. In 2022, approximately 2.5 billion adults worldwide were overweight, with 890 million suffering from obesity, while 390 million were underweight [1]. The health consequences of obesity are well documented, with numerous studies linking excessive fat to various diseases. For instance, apart from increasing the incidence of metabolic-related diseases (type 2 diabetes, hypertension, and stroke), obesity is also associated with the incidence and survival outcomes of certain cancers [2-4]. Currently, obesity has assumed epidemic proportions, and research is increasingly focused on assessing body fat distribution and measurement to monitor and mitigate the development of malnutrition-related diseases [5, 6].

Various techniques and methods are used to measure body fat mass (BFM) and assess nutritional status. The WHO recommends the use of body mass index (BMI) to detect obesity, defining overweight as BMI ≥ 25 kg/m^2^, and obesity as BMI ≥ 30 kg/m^2^. However, for Metabolically Obese Normal Weight (MONW) [7] and sarcopenic obesity (SO) [8], BMI may not be the optimal clinical parameter for assessing obesity [9]. While CT and MRI are considered the gold standards for quantitative fat measurement, especially intra-abdominal fat, concerns related to radiation safety and their high costs hinder their widespread use. Bioelectrical impedance analysis (BIA) and dual-energy X-ray absorptiometry(DXA), although capable of assessing BFM, are both influenced by the body’s hydration status [10]. Ultrasound has been widely applied in musculoskeletal assessment and has emerged as a promising tool for evaluating body composition [11-14].

Previous studies have used ultrasound to measure fat thickness (FT), but the consistency of measurement varies widely across studies [15]. In addition, the effect of body posture on measurement reliability remains largely unexplored, creating uncertainty in clinical protocols, especially for patients with limited mobility. Thus, this study aimed to fill this methodological gap by providing the first comprehensive assessment of how body posture affects ultrasound fat measurement reliability across multiple anatomical sites.

## 2 MATERIALS AND METHODS

### 2.1 Study Population

This was a cross-sectional study of 82 healthy volunteers recruited at the West China Hospital of Sichuan University from September 2023 to February 2024. The inclusion criteria were as follows [16]: (1) age over 18 years; (2) satisfactory compliance. The exclusion criteria were as follows: (1) pregnant women; (2) amputated arm or leg; (3) severe edema, indwelling metal pacemakers, or coronary stent; (4) any other reason that may prevent the individual from completing the study.

The study was approved by the Ethics Committee of the West China Hospital of Sichuan University (No. 2023[1828]). Written informed consent was obtained from all subjects prior to their enrolment.

### 2.2 Ultrasound Examination

The sonographic equipment used was the Mindray Ultrasound system (R9, Mindray, CHINA), equipped with an L14-3WU linear transducer and SC6-1U convex transducer. Abdominal visceral fat was measured using the “abdominal” default mode and the subcutaneous fat using the “general” default mode. The depth was adjusted according to the scanned FT. The frame rate was kept beyond 30 Hz.

Based on the studies by O'Neill *et al*. [17] and others [3, 18-20], FT was measured at the following ten sites across the body: site 1 (intra-abdominal FT), site 2 (pre-peritoneal FT, PPFT), site 3 (posterior right perinephric FT, PRPFT), site 4 (suprailiac FT), site 5 (abdominal subcutaneous FT), site 6 (anterior upper arm FT), site 7 (posterior upper arm FT), site 8 (subscapular FT), Site 9 (anterior thigh FT), Site 10 (posterior lower leg FT).

The procedure for ultrasound measurement involved localizing bony landmarks by palpation or ultrasound, locating the target sites with a soft ruler, and placing the probe laterally over the marked sites.

Examination postures were standardized to ensure accuracy. Sites 1-6 and 9 were scanned with the participant in the supine posture, and site 10 was scanned with the knee flexed to 90 degrees in the supine posture. Sites 7 and 8 were scanned with the participant in the sitting posture [16]. Throughout the procedure, participants maintained a standardized posture, keeping the right upper limb close to their trunk, with their palms facing forward. Special examination postures: Sites 7 and 8 required scanning in the prone posture, in addition to the routine posture. Similarly, site 10 was scanned in multiple postures: prone, left lateral posture, and seated, in addition to the routine posture. Photographs showing the various body postures are shown in Fig. (**1a**-**h**).

Throughout the scanning process, a sufficient coupling agent was used, and the probe was kept perpendicular to the skin without applying any pressure, allowing filling of the gap between the skin layer and the probe with the coupling agent. For measurement at site 3, volunteers were asked to inhale deeply, and the measurement was performed after obtaining a clear image. At the other sites, measurements were made under normal breathing. All sites were measured three times consecutively, and the average measurement was recorded in mm.

All ultrasound measurements were performed by a sonographer skilled in image acquisition. In our previous study, the consistency between repeated measurements in routine posture was demonstrated [16]. To ensure reliability in special examination postures, a second doctor with experience in the ultrasound department repeated the measurements for 30 participants.

### 2.3 BIA Examination

Body composition analysis was conducted using the InBody 270 device (Seoul, South Korea). The specific procedures were described in the previous study [16]. The BIA test was administered to each participant on a single occasion.

### 2.4 Skinfold Thickness Examination

Skinfold thickness was measured using a Digital Body Fat Caliper at sites 3, 7, and 8, with the participant in the standing posture. The specific procedures were described in the previous study [16].

To ensure comparability between different examinations, all examinations for a particular participant were completed on the same day.

### 2.5 Statistical Analysis

Statistical analyses were performed using SPSS 25.0 (IBM, Armonk, NY, USA) and GraphPad Prism10.0 (GraphPad Software, San Diego, CA, USA). Continuous variables were expressed as mean ± standard deviation (SD). The Shapiro-Wilk test was used to assess the normality of the distribution of continuous variables. The selection of statistical methods was based on whether the data to be compared was an independent or related samples. For normally distributed variables, the chi-squared test and *t*-test were used to compare two groups of independent samples, otherwise Mann-Whitney U test was used. Paired *t*-test was used for two paired samples. For non-normally distributed variables, chi-square test and Mann-Whitney U test were used to compare two independent samples, while the Wilcoxon Rank-sum test was used to compare two paired samples. The comparisons of FT at site 10 in four different postures were performed using the Friedman test, and the subsequent pairwise comparisons between two groups were also conducted using the Kruskal-Wallis test with adjusted P values. Pearson correlation analysis was used for normally distributed variables, and Spearman correlation analysis was used for non-normally distributed continuous and categorical variables. Intraclass correlation coefficient (ICC; Two-way Mixed Model, Absolute Agreement, Single Scores) was used to evaluate intra-observer and inter-observer measurement consistency at the sites. Two-tailed *P* values less than 0.05 were considered indicative of statistical significance. Sample size calculation was performed using PASS 15.0 software (NCSS, Inc., Kaysville, UT, USA). To ensure adequate statistical precision for the assessment of measurement consistency, which was our primary outcome measure, we set a 95% confidence level and a Width of Confidence Interval of 0.2. Based on pilot study data, we estimated the sample Intraclass Correlation Coefficient (ICC) to be 0.8. The minimum sample size we calculated was 55, so we will ensure that the sample size for the ICC analysis is greater than this value.

## 3 RESULTS

### 3.1 Demographic and Clinical Characteristics of the Study Population

A total of 45 males and 37 females were included in this study. The demographic and clinical characteristics are summarized in Table **1**. The mean age and BMI of participants were 23.0 ± 3.4 years and 22.4 ± 2.4 kg/m^2^, respectively. Male participants had a greater weight than female participants. There were no significant differences in age and BMI between males and females. However, males had lower body fat percentage (BF%) and body fat mass (BFM) compared to females.

### 3.2 FT in Routine and Special Postures

Table **2** shows the FT at each site in men and women in routine postures. There was no significant difference in FT between male and female participants at sites 2 and 3. FT at site 1 was significantly higher in men than women, and the FT at other sites was lower in men than women.

**Fig. (2):**
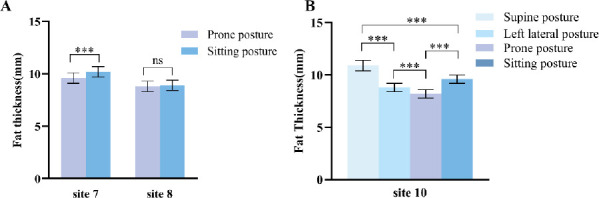
Comparison of fat thickness in different postures. (**A**) Comparison of fat thickness at sites 7 (posterior upper arm) and 8 (subscapular) in prone and sitting postures. (**B**) Comparison of fat thickness at site 10 (posterior lower leg) in supine, left lateral, prone, and sitting postures. *** *P* < 0.001; ns, no significant difference (*P* > 0.05).

We measured the FT at site 7 (posterior upper arm) and site 8 (subscapular) in the sitting and prone postures and found that the FT at site 7 measured in the sitting posture was significantly greater than that in the prone posture. However, FT at site 8 was not significantly different between the two postures. Detailed data are shown in (Table **3** and Fig. **2A**).

The FT at site 10 (posterior lower leg) was measured in four postures. FT was found to be significantly different in different postures. In the subsequent pairwise comparisons, significant differences were also noted between any two groups (*P* < 0.001). The detailed data are shown in (Table **4** and Fig. **2B**).

### 3.3 Analysis of Intra-observer and Inter-observer Consistencies

Table **5** presents the analysis of the consistency in special examination postures between repeated measurements performed by two measurers on 30 participants. The intra-observer ICC and the inter-observer ICC were both ≥0.925.

### 3.4 Correlations between FT and Skinfold Thickness

Analysis of the correlation between FT and skinfold thickness at sites 5, 7, and 8 revealed a strong positive correlation at all three sites. The details are listed in Table **6**.

## 4 DISCUSSION

This study demonstrates that ultrasound FT measurements exhibit good to excellent consistency and that changes in body posture influence FT.

Socioeconomic development and changing lifestyles have led to an increased prevalence of obesity in the general population. At the same time, many people are undernourished. Obesity and malnutrition are closely related to various diseases and have a significant impact on the quality of life. Therefore, finding reliable methods for measuring fat and monitoring nutritional status is a key research imperative.

Skinfold thickness is often used to monitor local FT, typically using calipers. It is a simple, low-cost, and easy-to-use tool that is usually the choice for screening large groups of people. Currently, BMI is one of the main criteria for judging the degree of obesity. However, BMI does not differentiate between fat and muscle mass, as individuals with a similar BMI may have completely different muscle-to-fat ratios. Moreover, individuals with normal BMI may have fat mass and body fat percentage exceeding the normal standard [6]. CT and MRI are considered the gold standard for quantifying body fat, especially abdominal fat [21, 22]. Although these modalities enable visualization of fat distribution and fat area, they are not suitable for screening purposes because of radiation exposure, high cost, and long examination time. BIA is a mainstream test for assessing obesity due to its convenience and simplicity of operation. It is increasingly being used in the general population and for academic research. DXA is an alternative test to BIA with a good correlation between the two [23]. However, both these investigations require specific devices, making them unsuitable for use by primary-level organizations. Moreover, their measurements are influenced by body water content.

In contrast, ultrasound is a safe and comfortable option for patients, making it a valuable tool for measuring FT. Störchle [24] demonstrated high measurement accuracy and reliability of ultrasound for FT measurement in trained hands. However, some studies have shown inconsistent reliability of ultrasound measurements [15]. Furthermore, the impact of various body postures on FT measurement accuracy remains an unresolved issue. Therefore, in the present study, in addition to analyzing measurement consistency, we examined whether the measurement differences in different body postures were meaningful.

This study demonstrated good reliability of ultrasonic FT measurement in special examination postures. These findings are consistent with previous research, which also showed high reliability in routine postures, with all intra-observer and inter-observer ICCs ranging from 0.610 to 0.996 [16]. All intra-observer and inter-observer ICCs were greater than 0.6, a statistically good agreement. Specifically, the subcutaneous fat sites had excellent repeatability regardless of posture (ICC>0.9), consistent with previous studies [25]. The measurement consistency at site 3 (PRPFT) has differed in previous studies. Ricci *et al*. [20] reported PRPFT of 13 ± 3 mm (ICC: 0.51), while Hazem *et al*. [3] reported PRPFT of 12.4 ± 1.7 mm, with the lowest ICC of 0.895. The PRPFT in Hazem's and Ricci's studies was significantly greater than in the present study (5.0 ± 1.6 mm). This may be attributable to their older study populations. Some studies have suggested an age-related increase in the deposition of fat in the abdominal cavity. Moreover, the subjects in the studies by Hazem *et al*. and Ricci *et al*. were of different races. European and American races generally have a greater BMI and higher BFM. Further studies should include a larger study population comprising different ages and BMIs to investigate the repeatability of PRPFT measurements [3, 20].

There is a paucity of research exploring the influence of body posture on FT measurements. Byenfeldt *et al*. [26] demonstrated that body posture affects liver attenuation parameters, yet found these measurements to be somewhat interchangeable across postures. Similarly, our study revealed that FT measurements remain largely interchangeable despite changes in body posture. In particular, site 8 (subscapular) showed no significant difference between the two postures. In other words, when it is inconvenient or inappropriate for the person to adopt a certain posture, a replacement posture can be used for measurements. FT measurements at sites 7 and 10 differed when the body posture was changed. FT at site 7 measured in the sitting posture was greater than that measured in the prone posture. For site 10, FT measured in all four postures varied, with the largest measurements being obtained in the supine posture, which was also the simplest posture to operate. However, we did not analyze the correlation of the data obtained in different postures with the gold standards for assessing fat tissue, such as CT and MRI. Therefore, we could not identify the specific posture that provides more accurate data. Furthermore, we found no significant difference in BMI between the sexes, but women had significantly higher BF% and BFM than men, suggesting that BMI may not be accurate enough for assessing body composition, consistent with previous studies [9]. In this study, women were found to have higher body fat, which was reflected in the greater FT at most sites in women. There were some exceptions. FT at site 1 was greater in males than in females. This sex-specific difference in fat distribution, with men predominantly accumulating visceral fat and women accumulating subcutaneous fat [27], may be attributed to the regulatory role of sex steroid hormones in adipose tissue distribution [28].

Skinfold is widely used to estimate local FT and BFM due to its ease of use in various settings. Our study revealed a strong positive correlation between local FT and skinfold thickness at corresponding sites. This supports the findings of Fanelli and Kuczmarski, who suggest that skinfold thickness and ultrasound measurements are equally valid for assessing body composition [29]. However, skinfold measurements have limitations. Variability in operational technique and measurement site can compromise the stability and consistency of results [18].

### 4.1 Strengths and Limitations

Ultrasound measurements of FT have proven reliable, offering advantages in cost, ease, accuracy, and scalability. Additionally, our study shows that data from different postures can be used interchangeably, making ultrasound highly practical. Ultrasound is particularly valuable in clinical settings where other methods for fat measurement, such as BIA, CT, or MRI, are impractical or impossible. For instance, ICU patients who cannot stand or be mobilized can benefit from ultrasound’s non-invasive and flexible assessment capabilities. It can enable accurate fat measurement and close monitoring of nutritional status. Furthermore, these results are particularly valuable for clinicians working with mobility-impaired patients, offering evidence-based alternatives to standard measurement protocols.

Some limitations of the study need to be acknowledged. Firstly, a major limitation of this study is the concentrated young participant pool (mean age 23.0 ± 3.4 years). The narrow age range limits the generalizability of our findings to other age groups. Age-related changes in fat distribution and tissue elasticity might affect ultrasound measurements differently in middle-aged and elderly populations. Therefore, our results should be interpreted with caution when applied to other age groups. Additionally, our sample comprised individuals with a relatively narrow BMI range. Future studies should include diverse age groups, a broader BMI range, covering various levels of obesity, and participants from different ethnicities.

## CONCLUSION

This study provides the first comprehensive evidence base for posture-specific ultrasound protocols in fat thickness measurement. Our results demonstrate that ultrasound is a reliable method for measuring fat thickness, exhibiting good to excellent inter-observer and intra-observer consistency. The impact of body posture on fat thickness measurements varies by anatomical location, with subscapular measurements remaining consistent across postures, while posterior upper arm and lower leg measurements show posture-dependent variations. Strong correlations were found between ultrasound measurements and skinfold thickness at subcutaneous sites, confirming the validity of ultrasound for fat thickness assessment.

Based on these findings, we recommend standardization of measurement protocols considering body posture effects. Ultrasound proves particularly valuable in clinical settings where traditional methods for fat measurement may be impractical or impossible, such as for patients with mobility limitations. This study had several limitations, including a concentrated young participant pool and a relatively narrow BMI range. Future studies should include diverse age groups and broader BMI ranges to establish comprehensive reference standards for clinical practice.

Overall, this research highlights the reliability and practical utility of ultrasound in accurately assessing body fat distribution, with important implications for nutritional status monitoring and clinical management of obesity-related conditions. The posture-specific insights provided can guide the development of standardized ultrasound protocols to improve the consistency and validity of body composition assessments.

## AUTHORS’ CONTRIBUTIONS

The authors confirm their contribution to the paper as follows: M.L.: Investigation; L.Q.: Methodology; Y.G.: Writing - Original Draft Preparation; X.T.: Writing - Reviewing and Editing Author. All authors reviewed the results and approved the final version of the manuscript.

## Figures and Tables

**Fig. (1):**
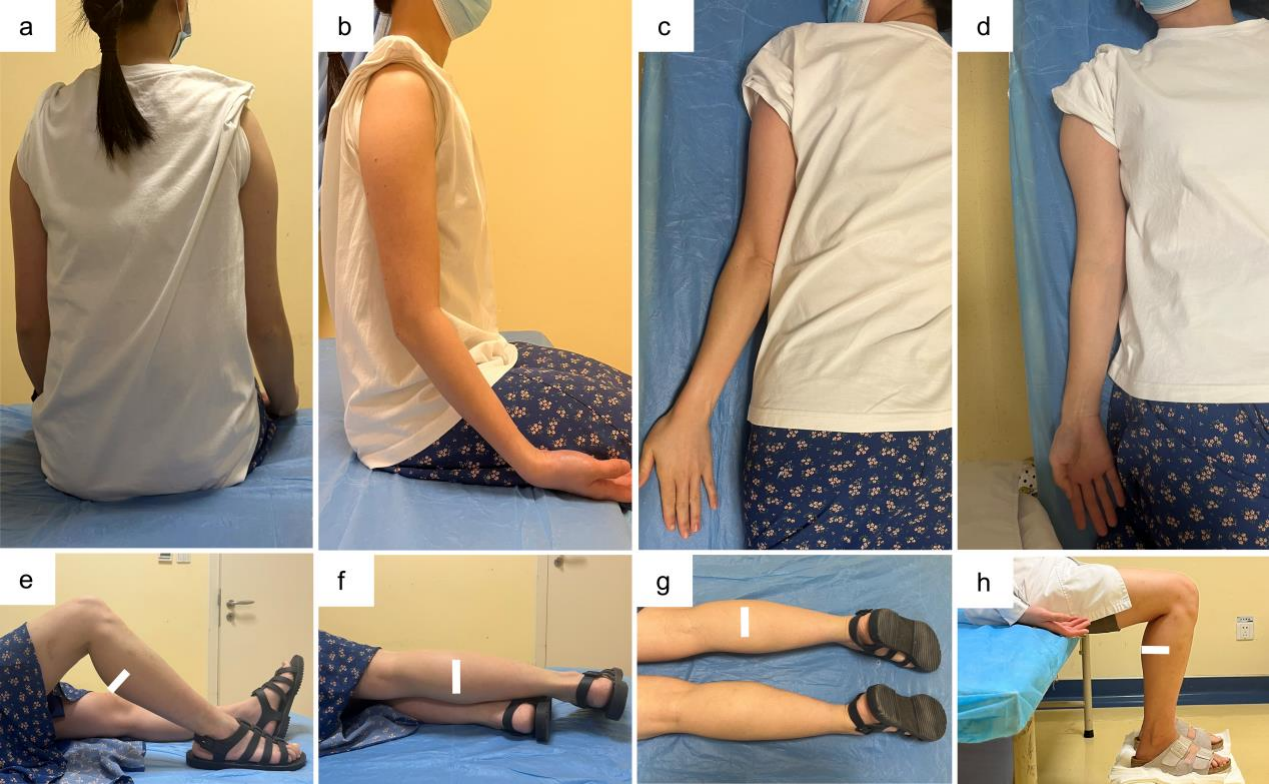
Representative photographs showing the postures of the participants. (**a**-**b**) Posterior and lateral views of sitting posture showing measurement at sites 7(posterior upper arm) and 8(subscapular); (**c**) Prone posture for sites 7 and 8; (**d**) Supine posture showing site 6 (anterior upper arm); (**e**-**h**) Lower leg measurements at site 10 in different postures: (**e**) supine, (**f**) left lateral, (**g**) prone, and (**h**) sitting posture.

**Table 1:** Demographic and clinical characteristics of participants.

**Parameter**	**Total (N=82)**	**Men (N=45)**	**Women (N=37)**	***P*-value**
Age (year)	23.0 ± 3.4	23.8 ± 3.9	22.1 ± 2.3	*P* = 0.046
Weight (kg)	63.8 ± 9.7	69.0 ± 8.6	57.4 ± 6.6	*P* < 0.001
BMI (kg/m^2^)	22.4 ± 2.4	22.9 ± 2.5	21.9 ± 2.3	*P* = 0.065
BF% (%)	25.2 ± 7.7	20.8 ± 6.3	30.5 ± 5.7	*P* < 0.001
BFM (kg)	16.0 ± 5.4	14.6 ± 5.7	17.8 ± 4.7	*P* = 0.005

**Note:** BMI, body mass index; BF%, body fat percentage; BFM, body fat mass.

**Table 2:** Fat thickness of males and females in the routine posture.

**Sites**	**Fat Thickness (mm)**		***P*-value**
	**Men (N=45)**	**Women (N=37)**	
Site 1	56.7 ± 8.4	46.2 ± 7.3	*P* < 0.001
Site 2	10.9 ± 4.2	11.1 ± 4.0	*P* = 0.795
Site 3	5.1 ± 1.9	4.9 ± 1.0	*P* = 0.780
Site 4	11.7 ± 7.0	16.3 ± 6.5	*P* = 0.001
Site 5	16.3 ± 7.9	22.6 ± 8.4	*P* = 0.001
Site 6	2.9 ± 1.6	5.7 ± 2.2	*P* < 0.001
Site 7	7.0 ± 3.1	14.1 ± 3.9	*P* < 0.001
Site 8	7.2 ± 3.8	10.9 ± 4.9	*P* < 0.001
Site 9	6.9 ± 3.1	12.5 ± 3.1	*P* < 0.001
Site 10	8.2 ± 2.7	14.2 ± 3.0	*P* < 0.001

**Note:** Site 1, Intra-abdominal; Site 2, pre-peritoneal, PPFT; Site 3, posterior right perinephric FT (PRPFT); Site 4, suprailiac; Site 5, abdominal subcutaneous; Site 6, anterior upper arm; Site 7, posterior upper arm; Site 8, subscapular; Site 9, anterior thigh; Site 10, posterior lower leg.

**Table 3:** Fat thickness at Site 7 and Site 8 in different postures.

**-**	**Prone Posture**	**Sitting Posture**	***P*-value**
Site 7 (mm)	9.6 ± 4.9	10.2 ± 5.0	*P* < 0.001
Site 8 (mm)	8.8 ± 4.8	8.9 ± 4.7	*P* = 0.289

**Note:** Site 7, posterior upper arm; Site 8, subscapular.

**Table 4:** Fat thickness at Site 10 in different postures.

	**Supine Posture**	**Left Lateral Posture**	**Prone Posture**	**Sitting Posture**	***P*-value**
Site 10 (mm)	10.9 ± 4.1	8.8 ± 3.5	8.2 ± 3.2	9.6 ± 4.0	*P* < 0.001

**Note:** Site 10, posterior lower leg.

**Table 5:** ICCs for intra-observer and inter-observer consistency of fat thickness in special examination postures.

	**Intra-observer**	**Inter-observer**
Site 7 in prone posture	0.994	0.925
Site 8 in prone posture	0.996	0.933
Site 10 in left lateral posture	0.990	0.950
Site 10 in prone posture	0.988	0.980
Site 10 in sitting posture	0.987	0.981

**Note:** ICC, intraclass correlation coefficient; Site 7, posterior upper arm; Site 8, subscapular; Site 10, posterior lower leg.

**Table 6:** Correlation between fat thickness and skinfold thickness at subcutaneous fat sites.

**-**	**R**		
	**Men (N=45)**	**Women (N=37)**	**Total (N=82)**
Site 5	0.828**	0.867**	0.921**
Site 7	0.874**	0.844**	0.878**
Site 8	0.890**	0.832**	0.882**

**Note:** Site 5, abdominal subcutaneous; Site 7, posterior upper arm; Site 8, subscapular; **, *P* < 0.01.

## Data Availability

The datasets generated and/or analyzed during the current study are available from the corresponding author [L.Q] on reasonable request.

## LIST OF ABBREVIATIONS

FT: Fat thickness
BFM: Body fat mass
BIA: Bioelectrical impedance analysis
ICC: Intraclass correlation coefficients
DXA: Dual-energy X-ray absorptiometry
PRPFT: Posterior right perinephric fat thickness
BMI: Body mass index

## STANDARDS OF REPORTING

STROBE guidelines were followed.
